# A new framework to consider equity in urban intervention planning, implementation and evaluation: development and application in a case study on an urban play spaces policy

**DOI:** 10.1186/s12889-026-26449-7

**Published:** 2026-02-24

**Authors:** Jenny Ahrens, Maddie White, Sonja Jeram, Gordana Ristovska, Jeroen Koning, Helene Gudi-Mindermann, Justus Tönnies, Gesa Czwikla, Miriam Weber, Gabriele Bolte

**Affiliations:** 1 Institute of Public Health and Nursing Research, Department of Social Epidemiology, University of Bremen, Bremen, Germany; 2 National Institute of Public Health, Ljubljana, Slovenia; 3 Institute of Public Health of the Republic of North Macedonia, Skopje, North Macedonia; 4 Faculty of Medicine, Ss. Cyril and Methodius University, Skopje, North Macedonia; 5 City of Utrecht, Utrecht, the Netherlands; 6 Health Sciences Bremen, University of Bremen, Bremen, Germany

**Keywords:** Health equity, Environmental justice, Intersectionality, Intervention, Implementation, Evaluation, Logic model, Urban planning

## Abstract

**Background:**

Improving population health while addressing social inequalities is a key challenge for public health strategies, particularly in urban settings. Effective interventions must consider the unequal distribution of both beneficial and harmful environmental exposures, as well as differential vulnerabilities. The Health in All Policies (HiAP) approach emphasises the role of multiple sectors in shaping health outcomes. To ensure equity, interventions should be assessed for their impact on social inequalities in health, considering the dimensions of environmental justice. We therefore developed a framework, consisting of a logic model of the intervention process with an equity-lens, to incorporate equity aspects into urban interventions to prevent the creation or exacerbation of health-relevant inequities in the urban environment.

**Methods:**

The framework was developed through extensive literature searches, inter- and transdisciplinary discussions and with expert knowledge. In this paper, we demonstrate its applicability using the example of a case study focussing on planning and implementation of play spaces in Utrecht, NL.

**Results:**

Incorporating equity into practice is challenging and requires interdisciplinary efforts to bridge the gap between theoretical consideration of equity aspects and the actual realisation in an intervention process. Our comprehensive framework addresses all phases of urban interventions (initial situation, planning, implementation, evaluation), emphasises environmental justice, and incorporates the HiAP approach, making it a versatile tool for equity assessment across sectors. Applying the framework to the case study revealed previously overlooked equity aspects and highlights how thorough consideration of equity aspects in urban intervention planning, implementation and evaluation will promote health equity.

**Conclusions:**

The new framework consisting of a generic logic model and an equity-lens can be adapted to and specified for urban interventions to address equity-relevant issues. In the context of sustainable urban development, the framework is intended to support integrating social and health equity as fundamental principles for the planning, implementation and evaluation of interventions.

**Supplementary Information:**

The online version contains supplementary material available at 10.1186/s12889-026-26449-7.

## Background

Improving population health has long been the central goal of contemporary public health strategies at local, national and international levels. These strategies aim to address health inequalities between population groups while enhancing overall population health. However, implementing strategies that explicitly address social inequalities in health remains challenging [1, 2].

To reduce social inequalities in environmental health in an urban context, it is important to address exposure variation with regard to social inequalities in both beneficial (e.g. access to restorative spaces) and adverse environmental exposures (e.g. road traffic noise) as well as differential vulnerability to the health effects of these exposures (effect modification). In the case of interventions in the urban context, an important consideration is environmental justice, a guiding principle and approach to tackle environmental health inequalities, with the three interconnected dimensions of distributive, procedural and recognitional justice that collectively contribute to a just and equitable society [3]. Distributive justice refers to socioeconomic and sociodemographic inequalities in environmental exposures that may exist and are judged as avoidable and unfair. It asserts that environmental resources and risk factors need to be distributed fairly if environmental justice is to be attained [4, 5]. Procedural justice emphasises fair and transparent processes for decision-making and governance and focusses on the opportunities the public has to be involved in decision-making processes that relate to environmental risks [6, 7]. Finally, recognitional justice centres on acknowledging and respecting diverse identities, cultures and experiences by affirming the value of marginalised perspectives and identities [8, 9]. Addressing challenges to reduce social inequalities necessitates effective urban interventions that strive for equitable distribution of resources and opportunities in urban settings. An approach that is helpful in this regard is Health in All Policies (HiAP). The HiAP approach as integrated governance to promote health emphasises that multiple sectors – in particular those outside the health sector (e.g. urban planning) – play an important role in shaping population health and health inequalities [10]. These sectors can contribute to reducing inequalities, but may also unintentionally increase them in a worst-case scenario.

Unintentionally increasing health inequalities in response to a policy or an intervention, so-called intervention-generated inequalities (IGIs), are of specific concern when considering social inequalities in environmental health. IGIs can result at any stage of the intervention process related to the planning, provision, delivery and the response to an intervention [2, 11]. An intervention increases inequalities if it disproportionately benefits population groups with lower risk of poorer health outcomes, for example those with a higher income or a better educational background, or is particularly harmful for population groups with a higher risk of poorer health outcomes, e.g. those living in a deprived neighbourhood. Moreover, an intervention implemented in an urban environment is part of a broader complex system and may interact with other elements and processes of that system [12]. Improvements in the quality of the residential environment may lead to rising rents which in turn may result in less affluent residents being displaced. For example, a recent study showed that urban greening was associated with subsequent gentrification processes in cities from Europe and North America [13]. Cole et al. [14] suggested a “modern urban environmental justice riskscape framework” to understand the transition of environmental exposures and health effects and how urban renewal processes may lead to gentrification and exacerbation of urban health inequity.

It is important to assess to what extent social inequalities are considered throughout the development and implementation of interventions and whether an intervention might entrench those social inequalities in health that are considered avoidable, unjust and unfair (‘health inequities’ [1, 5]). In doing so, an intersectionality perspective is essential to understand the interaction of several individual and contextual social dimensions as well as the systems and structures of power, privilege or disadvantage [15, 16]. According to the recommendations of Presseau et al. [16], it should be considered already during planning and implementation phases how interacting social identities and social structures could influence the effects of an intervention. Exploring potential differences in an intervention’s effects across a population is critical to understand whether and how the policy design and implementation process can be improved to achieve greater (health) equity and prevent IGIs.

### Aim

This work is part of the EU-funded exposome project Equal-Life [17], which has as an objective informing and sensitising stakeholders involved in policy making and municipalities about issues of environmental justice. In science-policy transfer in this project, we are aiming to increase awareness about health equity in relation to interventions/policies targeting the improvement of the urban environment, where there is an interplay of exposures from the societal, social, natural and built environment relevant for people’s health and wellbeing [17, 18]. In the sense of exposome research, the investigation of the totality of exposures, urban interventions comprise all kinds of interventions addressing environmental exposures of the external physical and social domains within a city. We developed the framework as a tool to support the embedding of a health equity-perspective in all phases of the urban intervention process, specifically planning, implementation and evaluation to prevent the intervention-generated creation or exacerbation of inequities. The generic framework consists of a logic model depicting the schematic process of an intervention and an equity-lens comprising equity-related questions, and can be adapted to different case studies or contexts (intervention-specific framework). The aim of this paper is to present the development of this novel framework and the demonstration of its applicability in one case study on play spaces in the city of Utrecht, the Netherlands (NL).

## Methods

We used a two-stage approach to develop the framework. In stage one, an exploratory literature search, inter- and transdisciplinary discussions within the Equal-Life network as well as the preliminary development of the generic framework and the preliminary specification as an intervention-specific framework for four different case studies were conducted. In stage two, for further improvement of the generic framework, a specific literature search and compilation of expert knowledge were performed. Additionally, the intervention-specific framework was applied and further specified for one of the four case studies from stage one, also through additionally integrating experience from practice.

### Stage 1: development of the framework

#### Initial draft

The first step of the development of the framework consisted of an exploratory literature search in May 2022 on logic models and frameworks as well as how urban health and equity aspects have otherwise been considered in environmental interventions. Based on this information and informed by previous own research on equity impacts of interventions in other areas of public health policies [19, 20], a first draft of the logic model was created. This generic logic model represented the scheme of an urban intervention while considering potential entry points for equity aspects within the different phases. Equity-related questions and prompts were created based on knowledge from the literature and intended to be used alongside the logic model as an equity-lens.

#### Inter- and transdisciplinary discussion

The initial draft of the generic framework, consisting of a generic logic model and an equity-lens, was presented and discussed in interdisciplinary scientific meetings of the Equal-Life project as well as the wider European Human Exposome Network in 2022. The draft was also presented and discussed transdisciplinarily during a co-design workshop in September 2022 and a stakeholder forum in November 2022 with stakeholders and members of the Equal-Life project to gain insights from practice. These activities brought together professionals from scientific organisations and from administration in the domains of mental and environmental health as well as urban planning. Policy makers and representatives of non-governmental organizations were slightly represented. Laypeople were not involved.

#### Development of preliminary intervention-specific frameworks

Within the Equal-Life project, four case studies of urban interventions in different European cities were selected and used to elaborate the generic framework into intervention-specific frameworks. A summary of these four case studies is provided in Additional file 1. The generic framework was applied in each case study by the authors (JA, MWh, SJ, GR, GB) to assess its applicability. For example, for the Play Spaces case study, which aimed at adequate and safe play areas for all children in Utrecht, NL, the lead policy makers involved in the planning, implementation and evaluation of this intervention shared their knowledge and experience from practice to better understand the process and components of the intervention.

### Stage 2: refinement of the framework

#### Literature search

In the second stage of development of the generic framework, we conducted a more specific literature search in the form of a scoping review to screen specifically for other frameworks that integrated an equity perspective and were developed for the field of urban planning and urban interventions. The objective of this literature search was to identify which equity-relevant issues are represented in research and review papers of urban interventions, and specifically how these have been incorporated into frameworks and other concepts intended for practical application by policy makers. PubMed and Scopus were used with two blocks of search terms (one relating to equity, the other relating to frameworks or concepts, both appearing in the Title and/or Abstract, Additional file 2). The searches were limited to English language. The search was performed in May 2024 and there were no limitations regarding the publication date. We obtained 1265 hits in PubMed and 4353 hits in Scopus. Inclusion criteria at the level of title/abstract screening were (i) presence of relevant keywords on equity and on framework/concept, (ii) peer-reviewed article and (iii) relating to humans. Full-texts were then reviewed and the reference lists of these articles were checked for further relevant literature. Additional file 3 summarises the 11 publications included in the scoping review and synthesised for the potential refinement of the equity-lens. This literature evaluation enabled us to refine the equity-lens that we proposed in Stage 1.

#### Further integration of expert knowledge from research and practice

To identify gaps or potentials for further improvement of the generic framework, comments from practitioners involved in two of the four case studies (Play Spaces Policy and Mobility Hubs, City of Utrecht) and researchers from the fields of children’s and/or community health, urban transport, environmental justice and/or participatory research were collected in structured discussions. Topics included were equity in urban (planning) interventions, participatory research, knowledge of generic aspects of logic models or similar tools and frameworks with an equity perspective, as well as specifically regarding our framework with the logic model and the equity-lens. The main findings concerning potential changes or improvements to the logic model and the equity-lens in our generic framework were summarised by one author and discussed with the co-authors within the research group. This finalised framework is presented in the results chapter of this paper.

### Application of the framework

#### Intervention-specific framework: Play Spaces case study

The intervention-specific framework from Stage 1 for the Play Spaces case study (logic model and equity-lens) was further refined and is presented in the results chapter of this paper. The Play Space Policy introduced in 2022 by the Municipality of Utrecht, NL, aims to provide suitable opportunities for play and exercise for young people in the densely-populated urban environment. It focusses on the provision and standard of public play space in the city for children and young people with a particular focus on 5-12-year-olds. This policy is in alignment with Utrecht’s spatial strategy for 2040 and the city’s objectives to ensure healthy urban living for everyone. Play space in the policy is two-fold; it focusses on larger public play spaces that can be used for play and exercise in neighbourhoods, such as purpose-built playgrounds, as well as playable green space and public space in the neighbourhood (e.g. free sidewalk space, car-free streets). The central component of this policy is an update of Utrecht’s play space standard from 2009 that provides the basic assessment of qualitative and quantitative criteria in the form of a so-called ‘neighbourhood scan’ to assess the availability and suitability of play space in individual districts and if it is within a walkable distance of children’s homes (e.g. reachable within a 200 m or 400 m walk).

The central proposition for this case study was to review the Play Space Policy in terms of how it already considers equity. Because the Play Space Policy comprises both the play space standard (including its concrete qualitative and quantitative criteria) and the process of implementing the neighbourhood scan, both aspects were reviewed for how equity aspects were already incorporated in the policy. A document review was performed for the Utrecht Play Space Policy with a focus on the equity aspects that were already included. In addition, the neighbourhood scans were reviewed. A draft of the intervention-specific framework for this case study was presented and improved based on input of one of the co-authors (JK) working at the Municipality of Utrecht with specific expertise on the Play Space Policy.

In order to gain further insights into equity aspects of interventions related to play spaces in general, a rapid review was conducted which comprised two different literature searches with the focus on equity and play spaces. Firstly, a systematic literature search in Scopus for international, peer-reviewed literature was conducted. The complete search strategy can be found in Additional File 4. To be able to also consider grey literature on this topic, a second search focussed on play spaces policies in Germany as an example. For this search, specific German keywords (e.g. “Spielleitplanung”) were used. Germany was selected as an example due to the existence of the integrative municipal planning tool “Spielleitplanung” with the focus on the needs of children and adolescents. The intervention-specific framework which we then further developed as a result allowed us to make suggestions about how to strengthen and expand the equity perspective in the implementation and evaluation within each district.

## Results

This chapter starts with an introduction to the generic framework consisting of the logic model and the equity-lens. The second part presents the results of the application of the framework to the Play Spaces case study to illustrate how the framework can be used in practice.

### Framework to incorporate equity aspects into context assessment, intervention planning, implementation and evaluation

**Fig. 1:**
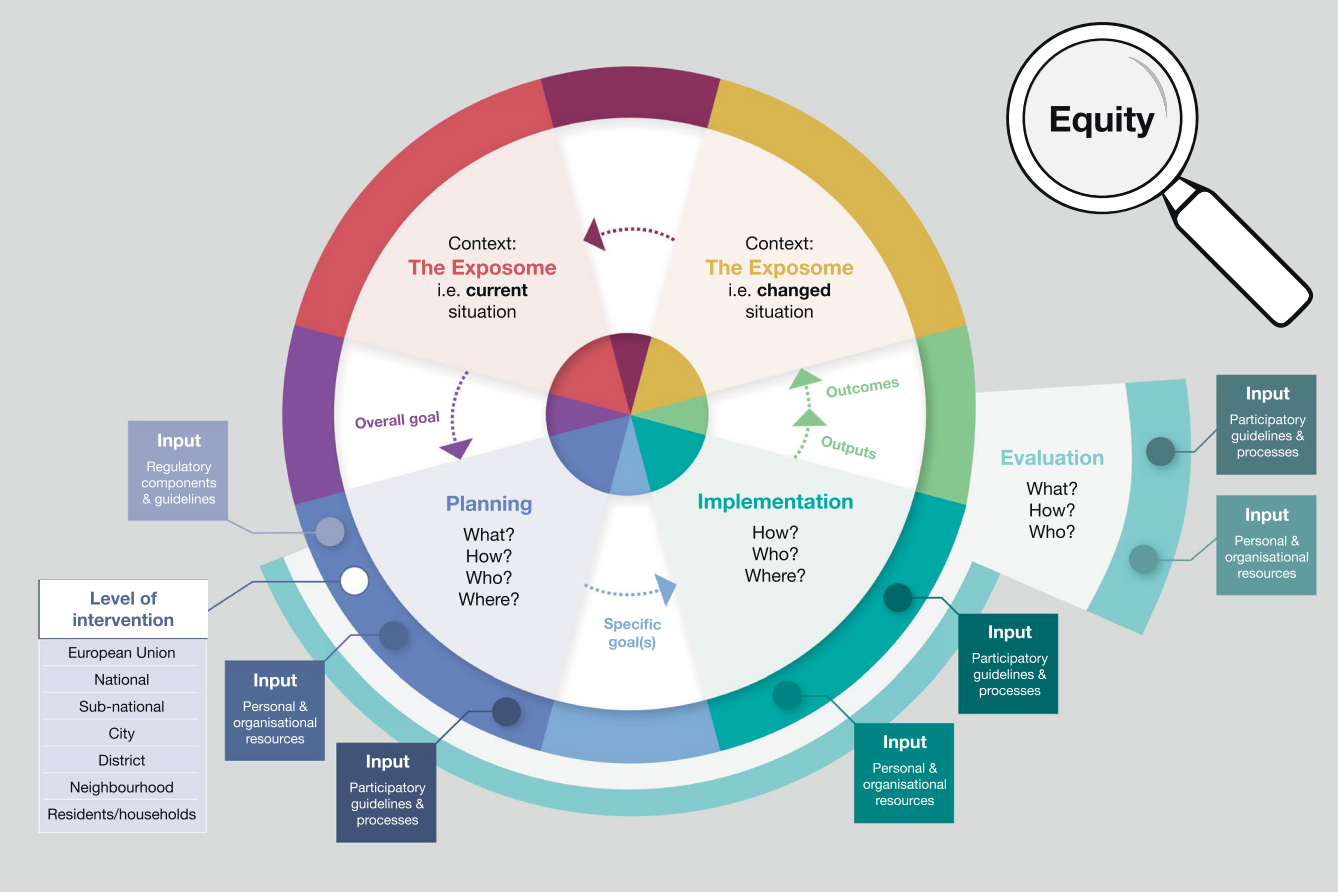
Logic model of the generic framework with the phases context, planning, implementation and evaluation, and an indication of the equity-lens

Within the logic model (Fig. 1), the intervention process is depicted schematically starting from the top-left and going counter clockwise. Although the key elements and the main phases of context, planning, implementation and evaluation are corresponding to the structure of a policy cycle, the more extensive elaboration with an equity focus implies a logic model. As such, the ‘bigger picture’ of this policy cycle, as depicted by the logic model, can be thought of as a three-dimensional spiral that moves closer and closer to health-promoting and equitable urban environments for all.

Table 1 illustrates the equity-lens of the generic framework (see columns 1–3) and its applicability to the Play Spaces case study (see column 4). The equity-lens comprises a selection of equity-related questions organised using the dimensions of environmental justice. It is a tool to conduct a compliance check regarding distributive, procedural and recognitional justice for interventions and/or policies. This lens is provided to factor in a broader equity perspective throughout the intervention process. These questions are intended as prompts to ensure that equity-relevant aspects are consistently included throughout the intervention process in order to prevent IGIs. Supporting information and recommendations are provided for each question to facilitate the use of this framework as a tool for policy-makers in practice.

**Table 1:** Equity-lens—questions as guideline to assess equity considerations in an intervention or policy

**Context**			
**Environmental justice dimensions**	**Questions relevant from an equity perspective**	**Supporting information and recommendations**	**Application in the case study: Play Spaces, Utrecht, NL**
Distributive Justice	What are the potential drivers of social inequalities in exposures that may not be immediately apparent?	• Consider characteristics like gender, age, disability, migration/indigenous status, education, employment/income, and their potential interaction (intersectionality perspective: multiple interlocking systems of power, privilege or disadvantage). • Additionally, contextual factors linked to place of residence or neighbourhood and societal processes such as discrimination related to culture and/or age. • Consider already ongoing processes of gentrification, which may be caused by previous place-based interventions.	*What was done:* Consideration of less apparent drivers: the interaction of characteristics, e.g. disability and higher age, migration background and low education level; and also pathways, e.g. low education and its impact on employment and income which leads to a life in poorer neighbourhoods and living in social housing, i.e. leading to potentially less access to private play opportunities. *Next step:* Consideration of other potential drivers such as discriminatory housing policies leading to differences in access to quality housing and neighbourhoods.
Distributive and procedural justice	Are there specific groups experiencing disadvantage due to structural factors?	• E.g. children or women living in poverty, elderly, people with disabilities, ethnic minorities, indigenous people.	Low-income residents, immigrants, refugees, elderly, children with disabilities, ethnic and/or cultural minorities.
	Are there ongoing or expected processes which may interact with a new intervention and lead to intervention-generated inequalities?	• Consider processes such as gentrification and displacement of disadvantaged residents.	Safety issues in disadvantaged neighbourhoods make parents not allowing their children to play far away from their home. While in the play space policy 200 m were used, which can be further than parents allow.

**Overall goal**			
**Environmental justice dimensions**	**Questions relevant from an equity perspective**	**Supporting information and recommendations**	**Case study: Play Spaces, Utrecht, NL**
Distributive, procedural and recognitional justice	Are there social inequalities in the current situation for the exposure(s) of interest?	• Assess the socioeconomic and sociodemographic structure and social milieu (including cultural aspects, e.g. norms and values) of the residents. Are those residents experiencing higher socioeconomic disadvantage, for example, also subject to higher exposure to negative environmental exposures and/or reduced exposure to beneficial environmental exposures? • Consider the opinion of representatives of all socioeconomic, sociodemographic subgroups and different social milieus on the intervention issue and their ideas on solutions of this issue.	*What was done:* For access to and reachability of play and/or green space, examples include: • disadvantaged neighbourhood with a lack of accessibility to a private garden as well as limited financial and time-related opportunities to participate in recreational activities (e.g. sports clubs); • limited access to mobility options and lack of financial resources, e.g. for day or weekend trips or vacations; • lower quality of play space (e.g. trash, broken equipment); • conflict of interest and use (children vs. teenagers).
	Does the overall goal imply to reduce social inequalities in environmental conditions? What are the assumptions and their theoretical underpinning about possible mechanisms for achieving the goal?	• E.g. overall goal to reduce proven social inequalities in detrimental environmental exposures versus overall goal to reduce the average exposure in a specific region. • Consider previous work on theory of change for urban interventions and evidence about mechanisms. • In disadvantaged neighbourhoods, the amount of activities, youth workers and social workers around playgrounds are intended to promote playgrounds and connect with people in these areas.	*Overall goal:* To provide suitable opportunities for play and exercise for all young people within walking distance in the densely-populated urban environment of the city of Utrecht. This may imply a difference in amount of play space and the quality by creating more and better playgrounds in disadvantaged neighbourhoods and/or neighbourhoods where houses do not have a private garden.
Procedural and recognitional justice	Who is involved in identifying the potential current issue/problem?	• E.g. top-down (problem identification by administration etc.) vs. bottom-up approach (problem identification by citizens; consideration of socioeconomic status or deprived vs. affluent neighbourhood).	*What was done:* No quantitative data, but qualitative “signals” on play space through key stakeholders and contact to key people from the neighbourhoods.
	What is the current situation in terms of the community’s health- and engagement-related capabilities (including health literacy) to understand the importance of the issue and to be involved in participatory processes?	• If available, use data on factors that can influence health literacy and capabilities, e.g. language (barriers), education, cultural differences, digital literacy, experience of discrimination.	*What was done:* Data from routinely sent out questionnaires (every 2–4 years) on community’s health literacy is available. Questionnaires are used to monitor the wellbeing of Utrecht’s citizens overall: Dutch language skills/language barriers, digital capabilities (e.g. use of computers), education, access/usage of healthcare. *Next step:* Using the knowledge from the questionnaires for participatory methods on problem identification.
	What is the current situation in terms of the community’s trust in relevant local authorities? How might this impact their willingness to be involved in participatory processes and what can be done to increase their faith in the process and encourage a meaningful involvement?	• If available, use experience or feedback from past projects or interventions. • Additionally, reach out to relevant stakeholders, neighbourhood organisations, etc.	*What was done:* Qualitative “signals” through key stakeholders and key people from the neighbourhoods providing feedback about the residents’ sentiment towards the municipality from past projects.
	How can exchange with the community be facilitated from an early stage?	• Identify who should be involved to facilitate community involvement. • Clarify what role the community plays during the intervention process e.g. feasibility of co-designing. • Clarify the extent of the planned participation based on e.g. the participation ladder [21].	*What was done:* Municipality of Utrecht: routinely checking different aspects that could lead to inequalities within the City of Utrecht using quantitative data: How many play spaces and children are in the respective neighbourhoods? *Next step:* If a need for improvement is assessed from the data: What participatory processes are needed to improve that?
	What participatory processes can be done to include people and how can different groups be reached and enabled to participate in the planning and the implementation of the intervention?	• Aim for co-designing and community-based participatory planning. Reaching different groups through contact and collaboration through different neighbourhood organisations, etc.	*What was done:* Surveys sent to principals and/or teachers of schools, surveys posted online for the residents, meetings and workshops with professionals and people in the neighbourhood, children were questioned if present at the playgrounds. *Lessons learnt:* Contacting schools in person worked better for getting feedback compared to only sending surveys online. *Next step:* involvement of residents from the neighbourhoods and schools (workshops with children or surveys for teachers to ask in class).
	What outcomes are important to residents?	• Assess this through e.g. co-designing the intervention with the community, community-based participatory planning.	*What was done*: • Opinions from professionals (key stakeholders e.g. youth workers, social workers, sports coaches) and other people in the neighbourhoods (“key figures”, resident representatives) who had submitted an initiative for new installations or improvements of a playground were included through a survey. • Meetings and in-person workshops were held in advance to discuss the play neighbourhood-boundaries. • In some districts the online survey was also sent to principals and/or teachers of some schools. • Whenever there were children present at the playgrounds their perspective was captured by asking them for their opinion on the playground and what they like and dislike about it. • For the evaluation of the playground scans in-person workshops are planned to include children’s opinions.

**Planning Who? Where? What?**			
**Environmental justice dimensions**	**Questions relevant from an equity perspective**	**Supporting information and recommendations**	**Case study: Play Spaces, Utrecht, NL**
Distributive and procedural justice	What data exists on the distribution of social inequalities in the area of interest?	• Conduct data analysis of the area of interest, e.g. with data on income, education, employment, migration, age, gender.	*What was done:* Data from City of Utrecht – Quantitative: population density, demographic structure in terms of age; – Qualitative: type of buildings (e.g. single-family home with garden or apartment buildings); – Others that would have been useful but were not at the right geographic scale: income, families living in relative poverty, education, health (e.g. life expectancy, obesity), children active in sports clubs.
	What social inequalities exist that may play a role in the implementation phase?	*Examples include:* – *Socioeconomic Status:* Do wealthier communities have more influence and are more active in decision-making processes? – *Language barriers and cultural differences:* Does everyone have access to the information about the intervention and do different cultural groups have different expectations regarding the intervention?	*What was done:* Consideration of: – Socioeconomic status: Wealthier communities having more influence and being more active in decision-making processes; – Language barriers and cultural differences: Everyone needs to have access to the information about the intervention, different cultural groups, even living in nearby houses/apartment blocks, have different expectations regarding the play space.
	What can be done in the planning of an intervention to address social inequalities that are known to exist in the implementation phase?	• Identify potential barriers and work to meaningfully anticipate and address them in the planning process. This includes identifying population groups that are important to be reached for the intervention to be successful.	*What was done:* For the prioritisation of the order of districts in which to conduct changes to the play spaces, the City of Utrecht based the decision on few socioeconomic and sociodemographic factors. During the process of creating the neighbourhood scans within each district, the supply (amount and type of the different play areas and playgrounds) and demand (in terms of population structure) of the play neighbourhoods was assessed. ‘Demand’ was assessed in terms of population density, demographic structure (age), type of buildings (e.g. single-family home with garden or apartment buildings) and experience of area professionals using aggregated data at the level of each play neighbourhood. *Lessons learnt:* In addition to the socioeconomic and sociodemographic aspects (population density, demographic structure in terms of age, type of buildings) already included, further characteristics (e.g. families living in relative poverty, migration) should be considered for additional prioritisation for improvements of play areas.
	What can be done in the planning of an intervention to implement accompanying measures to prevent or at least mitigate unintended processes and possible negative intervention effects?	• Identify available measures at the level of the planned intervention (e.g. national or community level) to avoid processes of gentrification and displacement of disadvantaged residents, e.g. rent control.	Up to now, in the Netherlands the initial placement of disadvantaged residents in already disadvantaged neighbourhoods has been identified as the main concern.
Regulatory components and guidelines			
Distributive, procedural and recognitional justice	What do existing regulatory components and guidelines tell us about social inequalities in relation to the issue being addressed by the intervention?	• I.e. consideration of vulnerability differentials by specifying different limit values or recommendations for certain population groups (distributive justice).	*What was done:* Issue: Equitable distribution of play space across neighbourhoods; prioritisation of investments in more disadvantaged areas. Consideration of guidelines and documents: - Participatory guidelines - Inclusion and accessibility guideline (UN) - Sports/Physical activity action plan - Green space guidelines - Climate change adaptation guidelines - Public space guidelines - Quality and safety guideline for playgrounds - Equity guidelines by City of Utrecht* Notes: *This was a new guideline published during the project and was used for the improvements of play areas.
**Specific goals**			
Distributive, procedural and recognitional justice	Are there any specific outputs and outcomes for subgroups of the population (e.g. that differ with regards to sociodemographic and socioeconomic factors or social milieu) that should be achieved with the intervention?	• E.g. different preferences or needs based on age, gender, socioeconomic status, ethnicity, physical or mental (dis-)ability.	*What was done:* Consideration of different ideas of ideal playground equipment, e.g. gender differences; necessity of equitable access for children with different abilities; social cohesion and cultural exchange, e.g. for different age groups.
	Which needs are prioritised in the case of conflicting interests between subgroups or between guidelines?	• Ensure a transparent decision-making process.	*What was done:* City of Utrecht gave prioritisation to the needs/interests of subgroups that were using the play spaces less before (e.g. interests of girls)
	Do the specific goals imply to reduce social inequalities in environmental conditions? What are the assumptions and their theoretical underpinning about possible mechanisms for achieving the goals?	• E.g. specific goal to reduce proven social inequalities in detrimental environmental exposures versus specific goal to reduce the average exposure in a specific region. • Consider previous work on theory of change for urban interventions with comparable specific goals and evidence about mechanisms.	*Specific goal:* Play space can be reached within 200 m of every home. In disadvantaged neighbourhoods this can be less and the amount of funding used to build and maintain is more in these areas to promote health.

**Implementation How?**			
**Environmental justice dimensions**	**Questions relevant from an equity perspective**	**Supporting information and recommendations**	**Case study: Play Spaces, Utrecht, NL**
Procedural justice	How can it be ensured that people are aware of the intervention and can be reached by the intervention itself?	• Information dissemination about the intervention using multiple media in different languages and also simple language (e.g. social media, websites, newspaper, posters).	*What was done: -* *Next step:* Exchange with community for all important aspects and phases: have several information and feedback rounds, use channels for different groups of people (e.g. online vs. newspaper/posters), collaboration with neighbourhood organisations (e.g. Wijkbureau), public events, use of blackboards/noticeboards.
	*If the intervention allows for adaptations during implementation:* How can it be ensured that people feel they can give feedback?	• Examples include: having multiple feedback opportunities (e.g. newspaper, surveys, online presence, meetings, events), also allowing anonymous feedback, reaching minority groups through contacting “key figures” of the neighbourhoods, neighbourhood organisations or religious institutions.	*What was done: -* *Next step:* Multiple feedback opportunities (e.g. newspaper, surveys, online presence, meetings, events etc.) and allow anonymous feedback, reaching minority groups through contacting “key figures” of the neighbourhoods or using connections to religious institutions.
	How can it be ensured that this feedback is being incorporated (if the intervention allows for adaptations) or justified and discussed (in cases when it cannot be incorporated)?	• Have a transparent process. • Indicate recognition and value of the feedback. • Ideally, have additional time, staff and funding to adapt interventions with regard to received feedback.	*What was done: -* *Next step:* Using updates on websites and in newspapers for the transparency of the process, e.g. publishing survey results.

**Evaluation Who? How? What?**			
**Environmental justice dimensions**	**Questions relevant from an equity perspective**	**Supporting information and recommendations**	**Case study: Play Spaces, Utrecht, NL**
Distributive, procedural and recognitional justice	Who is reached by the intervention and what change has taken place for those the intervention was designed for?	• Evaluate through data collection using surveys, meetings and interviews etc. in the neighbourhood, schools and with “key figures” from the neighbourhood.	*What was done: -* *Next step:* Evaluate use of play space and perceived neighbourhood quality through observation, data collection through surveys and interviews in the neighbourhood, schools and with “key figures”.
	How does the intervention affect those that are not directly targeted/addressed by the intervention?	• Identify population groups that were not the target group but could still have been affected by the intervention.	*What was done: -* *Next step:* Assessment of effects on other age groups (e.g. adults) than the intended group of children: health/wellbeing (negative: noise; positive: more access to green space), social cohesion across generations, changes in property values, conflict of interest in use of play spaces (children vs. teenagers) or noise from different sources (children, cars etc.).
	What are the direct and indirect effects of the intervention and how can they be assessed, also in terms of equity aspects?	• Identify direct and indirect effects and indicate how they can be assessed, e.g. through surveys, interviews, observation, (health) screenings, feedback rounds, public events etc. • Allow for multiple opportunities for data collection (surveys, online presence, meetings etc.), ensuring that minority groups can be reached and any kinds of barriers to comprehension such as language are considered. • Consider possible equity implications of the direct and indirect effects and collect data accordingly. • Use or establish an integrated ongoing monitoring to capture unintended processes with negative consequences such as gentrification and displacement of disadvantaged residents over the medium to long term.	*What was done: -* *Next step:* Assessment of effects Direct effects: • use of play space (assessed through observations, surveys, interviews), • increased physical activity in children (assessed through surveys, observations), • improved overall health and wellbeing, increased social interactions between children and neighbours (assessed through surveys, observations, interviews), • more accessibility (assessed through surveys and feedback rounds). Indirect effects: • development of the community (assessed through interviews, surveys, analysis of data), • environmental impact (e.g. assessing air quality, green infrastructure – Urban Heat Islands), • property values, crime reduction (e.g. for youth) • unintended processes with negative consequences, such as gentrification.
	Is there a differential impact of the intervention in different population groups?	• Consider different population groups (e.g. based on socioeconomic position, age, gender, ethnicity, religion, physical or mental (dis)abilities) to identify if there are social inequalities in intervention effects. • Allow for multiple opportunities for data collection (surveys, online presence, meetings etc.) ensuring that minority groups can be reached and any kinds of barriers of comprehensions such as language are considered.	*What was done:* The Play Spaces Policy was mainly intended for children aged 5–12 years old. *Next step:* Potential improvements: A change of public space also affects other members of the neighbourhoods/city, e.g. teenagers, elderly people. Therefore, a suggestion would be to aim at a more diverse offer for several age groups. Especially teenagers should be addressed more, since the need for space for youth was also highlighted within Utrecht. Teenage girls are playing less outside, but it’s very difficult to change that. Also, especially for playgrounds designed for younger children, options for their parents or other supervisors (e.g. seating) should be available and are mostly a discussion because people living surrounding a play space do not want this because of fear of youths “chilling”. *What was done:* Inclusion and accessibility were often mentioned in Play Spaces Policy: For the play scans, only physical disabilities have been considered (e.g. wheelchair use) in terms of being able to access the playground and also the playability of the playground elements (e.g. swings). This was done through observation, the playground space itself and the components of the playground were evaluated on how accessible they are. Additionally, every neighbourhood has a maintained play space (with a contact person present during opening hours) with designated play options for children with disabilities. *Next step:* Potential improvements: Inclusivity was partly incorporated in the scans; however, this aspect should also be included within the participatory approach (e.g. inclusion of children with various impairments). In general, considerations of impairments other than physical (e.g. visual) should be made.

The first stage where equity is to be considered is the CONTEXT, i.e. the current situation of the environment/exposome (see Fig. 1 and Table 1). This analysis of the current situation may reveal an issue or a problem (e.g. increased densification within cities, violence, poverty) with respect to a specifically considered exposure in the urban environment (e.g. access to public green space) or several exposures (e.g. noisy green space, pollution of the neighbourhood) that should be addressed by an intervention. At this first stage, elements of the complex system of the environment/exposome and ongoing processes should be analysed for a timely recognition of entry points for negative developments such as gentrification.

An overall goal is then identified that should be achieved by an intervention. In determining an overall goal from the context, the ‘problem’, the situation to be changed, needs to be defined and this should consider the distribution of the exposure(s) of interest. In this early phase, it is also important to check who perceives something as a problem, i.e. on what basis it is decided that an intervention and what kind of intervention is necessary. Attention should be given to the assumptions and visions when identifying the overall goal of an intervention. Consideration can be directed toward the theoretical or ideological assumptions that may be shaping how the problem and goal are defined. It can also be examined whose vision of improvement is informing the intervention’s objectives and how the expected pathway of change is being conceived. To facilitate procedural justice, participatory approaches play an essential role throughout an intervention’s planning, implementation and evaluation phases, including this problem-identification stage. While it is certainly important that participation is extended to the relevant organisations and authorities within a municipality, critical from an equity standpoint is the (meaningful) involvement of local residents. Important equity-related questions to consider at the early stages of an intervention relate to highlighting and understanding social inequalities that exist in a given context (e.g. city), as well as the mechanisms underlying these social inequalities, such as problematic exposure differentials, failure to recognise relevant (e.g. socially disadvantaged) population groups, failure to involve all population groups in a participatory decision-making process, and previous processes of gentrification, relocation of residents and segregation. In case of various issues arising simultaneously or competing interventions that are to be implemented within a similar time frame, it is necessary to prioritise the sequence of their execution based on timing considerations. Further aspects of the time frame of an individual intervention are included in the personal and organisational resources of the respective phases, see e.g. the Planning Phase section.

Equity-related questions need to be considered in the PLANNING PHASE to also ensure that an equity perspective is carried through the rest of the intervention process. During the planning phase of the intervention, it needs to be decided *who* is being addressed by the intervention, *how* the intervention is planned and should be carried out, *where* it is taking place, and *what* the intervention envisages in terms of improvements. The question of *where* refers to the administrative level of intervention (e.g. national, regional, sub-national, municipality/city-level, community and/or district or neighbourhood level) as well as to the spatial location where the intervention should take place. At the planning stage, the level(s) of particular focus for a given intervention and what will be done at one or more of these different levels need(s) to be set. The question of *what* includes the consideration of the whole urban system with ongoing processes into which the intervention will be implemented and possible interaction effects of the planned intervention with other processes leading to unintended negative effects such as gentrification. This may also include appropriate accompanying measures to prevent or at least mitigate these possible negative effects. The choices of the administrative level and spatial location may have a substantial impact on equity in this regard.

All these questions of *who*, *how*, *where*, and *what* refer to the interventions’ theory behind and underlying assumptions about mechanisms how to change the situation and processes leading to equity impacts.

Inputs at the planning stage—and also at the implementation and evaluation stages—include personal and organisational resources, regulatory components and guidelines, as well as participatory guidelines and processes:

- Personal and organisational resources include staffing, money and time. Personal resources refer to personnel, but additionally to individual expertise, commitment and willingness to collaborate across sectors and to promote equity.
- Regulatory components and guidelines set the policy direction for the development of a given intervention. These can include both legally binding and non-legally binding documents that are relevant as a policy-scaffold for exposure-related ambitions (e.g. Environmental Noise Directive of the EU).
- Participatory guidelines and processes relate to the participatory measures taken to promote community engagement (e.g. forums, workshops, surveys, invitations for feedback). These guidelines and processes facilitate meaningful involvement in the decision-making processes of an intervention from everyday community members (e.g. the 2010 Utrecht Standard for Participation).

Within the planning phase, more specific goals of the intervention are defined. To keep equity in mind during the formulation of these specific goals, the equity-related questions in Table 1 under “Specific goals” could be addressed.

In the IMPLEMENTATION PHASE, it is about setting into action *what* was decided on for an intervention’s approach in the planning phase. The implementation phase refers to *how* and *where* the intervention is being implemented and also *who* is responsible for which specific activities and outputs. From an equity perspective, it is critical to consider the potential impact of specific intervention activities on reaching diverse population groups. At this stage, what matters is not only whether these groups are reached, but also whether the intervention provides meaningful added value for those it was designed to benefit. Equity-relevant considerations include e.g. aspects of inclusivity and efforts to reach and involve minority groups. These are detailed in Table 1 with regards to how the focus during the implementation of an intervention can be on equity.

The implementation of the intervention leads to certain outputs and outcomes. An output refers to all visible or tangible products that result from a concrete activity within the intervention, while an outcome describes the added value that a product or service as a result of the intervention creates for the population group in focus of the intervention (the target group). The outputs and outcomes affect the context, resulting in a new changed situation in terms of diverse exposures (exposome). This changed context may lead to decisions about future interventions for further improvements.

The EVALUATION PHASE refers to *what* is going to be evaluated about the intervention itself (e.g. process and/or outputs and outcomes) and possible intervention-generated inequalities as unintended consequences of the intervention, *how* this will be done, and *who* is going to be involved in this evaluation of the intervention. The *what* has ideally already been negotiated in a participatory way in earlier stages of the intervention, e.g. during the formulation of the overall goal. *How* refers to data collection and compilation, where, from an equity perspective, stakeholders and residents should contribute. The question of *who* relates to both the parties directly involved in the planning and implementation of the intervention and also the meaningful involvement of residents as participatory evaluation. In the evaluation phase, the effects of the intervention for all (both those directly and indirectly affected) can be evaluated. To be able to assess possible intervention-generated inequalities that may occur even some time after completion of the intervention, for example gentrification processes and associated negative outcomes such as the displacement of less affluent residents, an adequate duration of the evaluation phase is necessary. This may imply that an integrated ongoing monitoring has to be implemented to assess also medium- and long-term equity impacts if it does not already exist in the specific urban context.

### Application of the framework to a specific intervention: Play Spaces case study

**Fig. 2:**
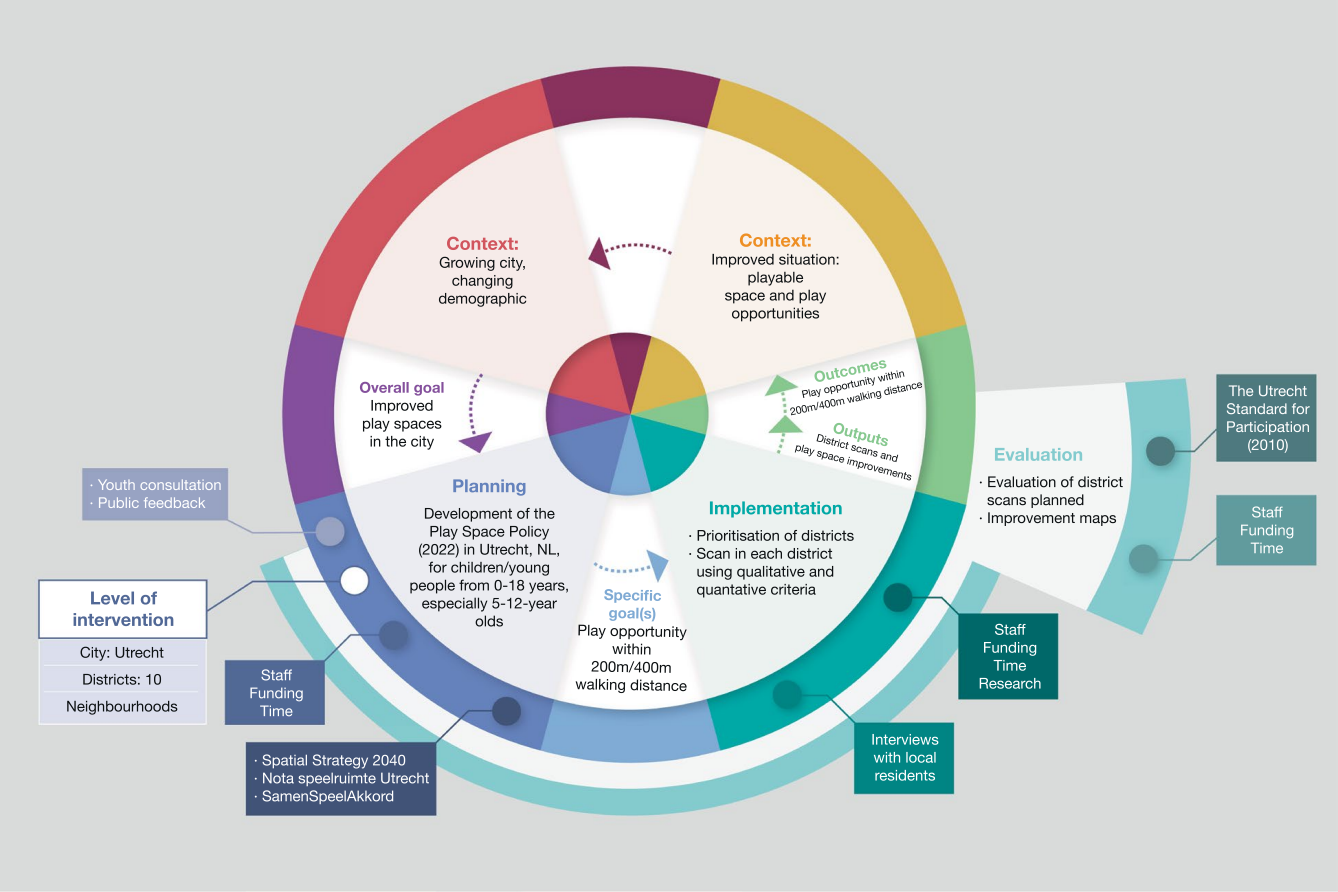
Logic model of the intervention-specific framework—Play Spaces case study (details given in Additional file 5)

The goal of the Play Space Policy in and of itself is to tackle social inequalities by aiming to ensure the Play Standard (play opportunity within walking distance) is met across the districts in the city of Utrecht and in the different play neighbourhoods for all children (with a particular focus on 5-12-year-olds). The Play Space Policy was investigated with the help of the framework consisting of the logic model and the equity-lens. The general outline of the case study is depicted in Fig. 2, while the figures in Additional file 5 give a more in-depth look into the planning, implementation and evaluation phases. What was done by the City of Utrecht, lessons learnt and what could be improved in order to ensure equity can be found in the last column of Table 1. Within the Play Space Policy, there are already some further aspects and considerations that are relevant from an equity perspective, e.g. prioritising the assessment of socially deprived neighbourhoods. While the logic model helped to get a (visual) overview of the intervention and to identify the most important information, the equity-lens of the framework helped to identify gaps that should be considered and addressed in the future, e.g. regarding participation, inclusion and accessibility as well as the consideration of different population groups from an intersectionality perspective. While participation was considered during all the phases of the Play Spaces case study, as it can also be seen in Table 1, further steps or improvements in participation as identified by the equity-lens could have been facilitated through more involvement of children and teenagers, instead of a main focus being on key people from the neighbourhoods and teachers or principals working in schools. For example, it turned out that children were particularly concerned with walkability, green spaces and parks, while adults seemed to be more concerned with parking and car issues when looking at the built environment.

Furthermore, targeted participation of children or families with first-hand experience with disabilities instead of observations could have helped to make a difference in terms of accessibility and inclusion. The play scans focussed exclusively on mobility-related physical disabilities, particularly regarding access to the playground (e.g. wheelchair use) and the usability of play elements (e.g. swings). Other impairments, such as visual or auditory disabilities, were not considered.

With regard to socioeconomic and sociodemographic aspects used to prioritise neighbourhoods, applying an equity-lens revealed that additional data could have improved the process – better identifying and targeting people in the most disadvantaged neighbourhoods. The Municipality of Utrecht based the decision on few characteristics of the resident population and districts (population density, demographic structure in terms of age, type of buildings).

## Discussion

This paper presents a framework to facilitate the incorporation of equity aspects into intervention planning, implementation and evaluation in urban settings in order to prevent IGIs and promote environmental justice. This framework consists of a generic logic model for interventions in the form of a visual representation as well as an equity-lens in the form of a structured compilation of questions that can be used as prompts by practitioners to embed an equity perspective into their work. The generic logic model is designed to obtain a systematic overview of all intervention phases and relevant inputs. The equity-lens of the framework (in the form of guiding questions) can be applied to the specific components of the logic model and identify the important equity aspects that need to be considered during the entire process from an environmental justice perspective. The generic logic model and the equity-lens can be used and further elaborated for specific interventions. In this paper we have demonstrated the successful application of the framework in the Play Spaces case study as an example of an urban intervention in the city of Utrecht.

The primary application we envisaged when developing the framework were interventions to improve the built, physical and natural environment in cities for healthier urban settings and specifically for universal interventions, which are designed for the general public. The framework can also be used, however, for interventions addressing the social environment as well as for non-universal interventions aiming to reduce health inequalities, e.g. targeting more specific groups or using a proportionate universalism approach [22]. In breaking down the intervention process into the different stages and highlighting potential entry-points for unwanted IGIs, the framework supports practitioners from various disciplines and sectors who are involved in urban planning projects to apply an equity-lens and critically reflect equity-relevant considerations during the whole intervention process.

Based on an extensive literature search, we identified models and frameworks in the field of urban planning and urban interventions pursuing similar goals as our framework [9, 23–32] (Additional file 3). Compared to these previous considerations or models for equity assessment, which often focus on a specific type of urban intervention such as improvement of air quality [23], green infrastructure [25] or transportation [27, 28], our framework is applicable to a variety of urban interventions, while still being comprehensive and detailed, which makes it a versatile tool for different disciplines. The equity-lens in our framework has a particular focus on environmental justice aspects, which is also seen in the framework of Meerow et al. [9] on urban resilience planning and in the framework of Okamato and Doyon [30] on urban coastal adaptation planning. However, in the majority of other models, environmental justice aspects were largely only mentioned indirectly and often only regarding distributional justice, while neglecting the dimensions of procedural and recognitional justice that are central in our framework.

The HiAP approach was an important starting point for the development of our framework by including a focus on different sectors, e.g. through regulatory documents. Cartier et al. [23] also refer to the HiAP approach in their work on interventions on air pollution in urban environments. WHO emphasises intersectoral action for health as one core element of the Urban Health Equity Assessment and Response Tool (Urban HEART) [32]. Additionally, the majority of the previous frameworks and concepts focus mainly on the planning phase of an intervention, while our framework focusses on all phases of an intervention (context analysis, intervention planning, implementation, evaluation) in order to cover relevant equity aspects of the different phases that may also occur after the planning phase. In the growing research area of implementation science, it has been repeatedly proposed to include an equity focus. This also means to take into consideration all aspects of the context, from the population or setting of the planned intervention to the macro-level forces that may influence the implementation of an intervention [33]. The equity-lens of our framework includes these aspects by considering structural processes of discrimination already in the context dimension, referring from an intersectionality perspective to multiple interlocking systems of power, privilege or disadvantage [15, 16].

Regarding the consideration of equity aspects in strategic environmental assessment in Italy, Lamorgese and Geneletti [29] observed that while equity is generally accounted for in the planning phase of urban interventions, there is often a gap in its consideration during the actual implementation process. For the assessment of equity of transportation systems and especially emerging transportation technologies, Guo et al. [27] emphasised the importance of interdisciplinary efforts to overcome research gaps. Our framework addresses these gaps, by adopting an interdisciplinary focus and considering all phases: context, planning, implementation and evaluation. Another strength of our framework is that by highlighting the potentially relevant data sources it clearly sets out the kind of information that is relevant from an equity perspective during the intervention process and can initiate or inform appropriate data collection processes to ensure that an equity-focussed evaluation is possible. Moreover, against the background of a potential entanglement of the implemented intervention with previous or ongoing processes leading to unintended consequences such as gentrification over the medium to long term, an integrated ongoing monitoring would form a basis for a comprehensive evaluation of all intervention-generated inequalities.

Our framework comes with some challenges. One part of the framework consists of a generic logic model as a visual representation of the aims, elements and processes of an intervention as well as the context [34]. The difficulty of such logic models in general is to balance simplicity and complexity and to take dynamic changes during real-world implementation into account [35]. The advantage of our generic logic model in being applicable to different types of interventions also has the limitation that for this purpose the logic model has to be generalised and simplified. When looking at a specific intervention of interest, a detailed examination of the underlying programme theory, the elements and processes of that intervention, relevant mechanisms and its outputs and outcomes is necessary [34]. Thus, in order to represent the complexity of an intervention process with the variability and multi-faceted options of intervention processes, a detailed addition to the model related to the specific intervention to be investigated is required. We have demonstrated the applicability of our framework initially in four case studies as part of the Equal-Life project and elaborated one of these case studies in this paper. Regarding the equity-lens, a difficulty when applying the framework is the potential limited availability of some data that is ideally needed to answer the equity-related questions. For the development of the framework, co-design activities comprised inter- and transdisciplinary discussions, but not engagement with the public. Future work with the framework and its further improvement should integrate knowledge and opinions of residents in co-design.

The framework should be seen as a starting point for collecting all important data directly in future processes of intervention planning, implementation and evaluation. It is an advantage of the framework that it is not static as it can also be initially filled with available information or data and gradually expanded during the process of the intervention as more data is collected.

A recent review on studies evaluating policy outcomes by using quasi-experimental methods showed that up to now logic models have been rarely used to improve conceptualisation of possible complex equity impacts and that the analysis of equity dimensions was limited [36]. Our framework combining a logic model of an intervention with an equity-lens is intended to integrate equity dimensions from the beginning to be able to assess and strengthen positive impacts of an intervention on equity and avoid negative unintended impacts in terms of intervention-generated inequalities. It complies with previous recommendations [25, 30] to define equity and to integrate distributive, procedural and recognitional justice dimensions. Thus, its application may help to maximise health equity in the context of urban interventions – an unmet need that has been repeatedly highlighted [23, 25].

Within the case study of the Play Spaces Policy in Utrecht, NL, the application of our framework has helped to identify gaps in important equity-related aspects. The main areas for potential improvement in terms of an equity perspective in the policy have been identified as participation, inclusion and accessibility, as well as the consideration of different population groups from an intersectionality perspective.

Participation with regard to this policy is critical not only from a general equity perspective as we have highlighted with the equity-lens and procedural justice dimension in our framework, but also specifically in the case of play space interventions. Involving playground users and community members has been described as an invaluable and under-utilised resource to maximise inclusion of all children in the playground environment [37] and has been found to have positive impacts on children, their use of parks, and their physical activity [38–40]. Children may provide important insights into the built environment [41]. Understanding children’s perspectives on playgrounds is necessary to gain a deeper understanding of the factors that determine children’s physical activity in playgrounds [42].

In the current case study, the participatory processes that were part of the play scan process included children and resulted in several ideas that would not have been considered by academics or other community members. However, it is also important to note that there may also be some limitations and barriers to the involvement of children and teenagers. It was noted by Winter et al. [43] that particularly in low-income ethnic minority communities, some children felt hesitant to get involved. Furthermore, it was commented by researchers that some of the children did not feel safe to participate in such in-depth investigations and to lead the process [44]. Thus, potential for further improvement lies in an even stronger involvement of children and teenagers instead of a main focus being placed on other people from the neighbourhoods and teachers or principals.

Further gaps identified in the application of the framework to the Play Spaces case related to issues of inclusion and accessibility. These topics were often mentioned within the Play Space Policy, but restricted in the play scans on physical disabilities in terms of being able to access the playground. In addition to obstacles for children with physical disabilities (e.g. inappropriate ground cover, lack of ramps, ropes, hand rails, ill-placed equipment and inaccessible routes) [45–48], also a diversity of ground-level components [49] would be beneficial. Merely having access to a playground is not enough to overcome exclusion and other emotional barriers [45]. Unsuitable playground design, meaning equipment and environment are not adapted to accommodate the children’s needs, can lead to social barriers, including prejudice and exclusion [48], which further entrench social inequalities.

As for the prioritisation of districts, the Municipality of Utrecht based the decision on few characteristics of the resident population and districts (population density, demographic structure in terms of age, type of buildings). However, further socioeconomic and sociodemographic characteristics of the resident population (e.g. families living in relative poverty) could be considered from an intersectionality perspective for additional prioritisation, especially for improvements of play areas. For example, evidence from Australia and the United States of America suggests that disadvantaged neighbourhoods (e.g. low income, low education, high percentage of ethnic minorities) tend to have fewer or lower-quality public open spaces compared to more advantaged areas. This may discourage their use and, in turn, limit opportunities to support young children’s mental health [50–52]. Furthermore, studies found that public open spaces may be more important for the mental health of children from socioeconomically disadvantaged backgrounds, since close proximity to green spaces reduces parenting stress especially in low educated parents [53, 54].

Youth of low socioeconomic status are more likely than their affluent peers to report that a nearby recreational facility is important to their level of physical activity, possibly because they have limited access to more distant (or more expensive) options [55, 56]. Findings in the Netherlands by Krishnamurthy [57] also showed that in areas with a lower socioeconomic position the children spend more time playing on the streets compared to a neighbourhood with higher socioeconomic standard. Based on this, it has been suggested that resources should be prioritised to and efforts focussed on low-income neighbourhoods to ensure that all children and adolescents have access to safe and appealing opportunities for play and active lifestyles [38, 58].

## Conclusion

By introducing a novel tool designed to embed a health equity perspective into context analysis, planning, implementation and evaluation phases of an intervention, our study highlights the need to acknowledge the central role of equity in urban interventions and to prevent intervention-generated creation or exacerbation of inequities. Urban interventions should ensure the equitable distribution of resources, opportunities and services in cities, addressing inequalities related to societal and political processes of discrimination related to social dimensions such as income, ethnicity or gender. A contribution to unintended processes of gentrification and displacement should be avoided. Additionally, recognitional justice and adequate participatory processes are crucial.

Our framework consisting of a generic logic model and an equity-lens can be adapted to and specified for certain urban interventions to highlight obvious as well as concealed equity-relevant issues. By applying the framework to the Play Spaces case study, this practical proof of concept provided insight into which equity aspects had remained unnoticed so far, could be unveiled with the framework and should be focussed on more. The intervention-specific logic model and the application of the equity-relevant questions as part of the equity-lens helped to critically reflect on the different phases and approaches within the intervention and to develop ideas on how to achieve more equity. Further research on the application of the framework to other urban interventions will help to illustrate how it can be used in practice to strengthen an equity perspective in policy work in urban settings.

While increasing urbanisation offers numerous opportunities for economic growth and social development, it may also exacerbate socioeconomic inequities, which, in turn, poses challenges for urban planners, public health practitioners and policy makers. In the pursuit of sustainable urban development, the concept of social and health equity needs to be a fundamental principle for the planning, implementation and evaluation of interventions. Equity in urban interventions is not only a matter of social justice; it is also linked to environmental sustainability and the overall resilience of cities.

## Supplementary Information

Supplementary Material 1. Overview of case studies. Table with basic characteristics of the four case studies.

Supplementary Material 2. Search terms. Search terms for the refinement of the equity-lens.

Supplementary Material 3. Overview of equity-related models and frameworks identified in scoping review. Table with main characteristics of the identified models and frameworks.

Supplementary Material 4. Search terms. Search terms for literature on play spaces.

Supplementary Material 5. Logic model of the intervention-specific framework for the Play Spaces case study, detailed for the phases planning, implementation and evaluation. Three figures showing details of the logic model for the Play Spaces case study, Fig. S1 planning phase, Fig. S2 implementation phase, Fig. S3 evaluation phase.

Supplementary Material 6. The Early environmental quality and life-course mental health effects (Equal-Life) project team.

## Data Availability

The datasets used and/or analysed during the current study are available from the corresponding author on reasonable request.

## Abbreviations

HiAP: Health in All Policies
IGIs: Intervention-generated inequalities
NL: The Netherlands
WHO: World Health Organization
