# Supplementary material for: A new framework to consider equity in urban intervention planning, implementation and evaluation: development and application in a case study on an urban play spaces policy
Source: BMC Public Health. 2026 Feb 24;26:721. doi: 10.1186/s12889-026-26449-7 (PMC12930818; doi:10.1186/s12889-026-26449-7)
Supplement: Supplementary file 1 — Supplementary Material 1. Overview of case studies. Table with basic characteristics of the four case studies. [file 12889_2026_26449_MOESM1_ESM.pdf]

# Additional File 1

## Overview of case studies

| Case Study | Noise Legislation | Cycle Infrastructure | Mobility Hubs | Play Spaces Policy |
|---|---|---|---|---|
| **Overall goal of the intervention** | Reduction of environmental noise exposure | Improvement of bicycle infrastructure and increase in cycling behaviour | Improving use of shared mobility and supporting transport accessibility, reducing transport poverty | Provision of adequate and safe play areas for all children |
| **Location** | Slovenia with a particular focus on the city of Ljubljana | Skopje, North Macedonia | Utrecht, the Netherlands | Utrecht, the Netherlands |
| **Intervention** | Implementation of Environmental Noise Directive 2002/49/EC and preparation of Noise Action Plans in Slovenia, and in Ljubljana in particular | Bicycle traffic improvement plan for the city of Skopje | Pilot study - shared mobility hub in Kanaleneiland neighbourhood | Play neighbourhood scans - assessment and improvement of play areas in different neighbourhoods in Utrecht |
| **Aspect of urban environment** | Physical environment | Built environment, transport infrastructure | Transport infrastructure, social infrastructure | Built environment, social infrastructure |
| **Publication** | Jeram et al. (2023) | - | Tönnies et al. (2025) | This paper |

Jeram S, Frigelj N, Kralj M, Ristovska G, Boljka U, Škafar M, et al. Implementation of the Environmental Noise Directive 2002/49/EC in Slovenia seen through an equity lens. 14th ICBEN Congress on Noise as a Public Health Problem. 2023. https://www.icben.org/2023/presenting193.pdf. Accessed 3 Aug 2025

Tönnies J, Ahrens J, Hasselder P, van Houten P, van Eerten JJ, White M, et al. Tackling transport poverty with offers for shared mobility – a qualitative analysis on mobility needs and barriers in the context of a new mobility hub in a deprived neighbourhood. Cities & Health 2025. https://doi.org/10.1080/23748834.2025.2539608
