# Supplementary material for: A new framework to consider equity in urban intervention planning, implementation and evaluation: development and application in a case study on an urban play spaces policy
Source: BMC Public Health. 2026 Feb 24;26:721. doi: 10.1186/s12889-026-26449-7 (PMC12930818; doi:10.1186/s12889-026-26449-7)
Supplement: Supplementary file 2 — Supplementary Material 2. Search terms. Search terms for the refinement of the equity-lens. [file 12889_2026_26449_MOESM2_ESM.pdf]

Additional File 2

Search terms for the refinement of equity-lens

Block	Topic	Search terms
1	Key words relating to equity	equity perspective, equity lens, equity-focus*, equity-oriented, equity-centred, equity-centered, equity impact*, equity effect*, equity issues, equity consideration*, equity concerns, equity implication*, equity analysis, equity measures, equity gap
2	Key words relating to a framework of some kind	Framework, model, concept*, tool*, assessment, evaluation, HEIA, theor*, mechanism*, pathway*, explanat*
