# Supplementary material for: A new framework to consider equity in urban intervention planning, implementation and evaluation: development and application in a case study on an urban play spaces policy
Source: BMC Public Health. 2026 Feb 24;26:721. doi: 10.1186/s12889-026-26449-7 (PMC12930818; doi:10.1186/s12889-026-26449-7)
Supplement: Supplementary file 3 — Supplementary Material 3. Overview of equity-related models and frameworks identified in scoping review. Table with main characteristics of the identified models and frameworks. [file 12889_2026_26449_MOESM3_ESM.pdf]

Additional File 3

Overview of equity-related models and frameworks identified in scoping review

Author/ Reference	Description	Stage(s) – (P)lanning, (I)mplementation, (E)valuation	Population(s) of interest	Exposure(s) of interest	Case studies	HiAP	Environmental Justice Aspects – Procedural (P), Distributive (D), Recognitional (R)
Cartier et al. 2015 [23]	Tool for assessing health and equity impacts of interventions and policies affecting air quality, with the main steps: emitting sources, pollutants, exposure, health effects.<br><br>Additionally, a set of questions divided into sources to pollutants, pollutants to exposure and exposure to health effects.<br>The target group are non-health experts, like urban planners and health policy decision makers.<br><br>The tool aims at the identification of modifiable (e.g. regulatory context, green spaces, behaviours), non-modifiable factors (e.g. age, neighbourhood, weather) as well as partially modifiable factors (e.g. socioeconomic status, comorbidities, architecture, demand).	P, E	General population in developed countries	Air pollution	Low-Emission Zone, Public bicycle sharing program	(x)	-

Cunha & Silver 2023 [24]	Planning support tool for assessing the relative equity impact of bicycle planning (TIRE); it provides a micro-scale spatial assessment of the effects of cycling infrastructure provision on the accessibility levels of distinct socioeconomic groups. The tool assesses the extent to which the cycling network allocation is equitable.	P, I, E	General population	Cycling infrastructure allocation	Application in Lisbon, Portugal	-	(D) = mention of distributive justice, in the context of cycling network coverage and accessibility favouring wealthier areas of the city while disregarding vulnerable and disadvantaged populations<br>(P) = mention of procedural justice and participatory approaches as a theoretical foundation of social impacts of transportation systems
Grabowski et al. 2023 [25]	General framework for examining the equity of urban planning applied to Green Infrastructure (GI) planning with the dimensions: Vision, Processes, Distribution in combination with a table "Plan Equity Evaluation Screen" evaluating based on the three dimensions with scores from 0 to 4.	P, I, E	General population in the US	Green Infrastructure	-	-	(D) = defined as one of the major dimensions of equity and examines the intended impacts in the categories of hazards, value, and labour<br>(P) = defined as one of the major dimensions of equity and includes the categories of how the plan itself is constructed, how GI is designed, how it will be implemented, and how its real-world impacts will be evaluated<br>(R) = highlighting the importance to integrate core principles of recognitional and transformative justice for urban planning

Gu et al. 2022 [26]	Framework with the dimensions density, diversity and design to measure the built environment around elderly communities; equity analysis is conducted from the social and spatial perspective.	P	Elderly community in Nanjing, China	Public amenities, road networks	Nanjing, China	-	(D) = mention of uneven distribution of amenities, e.g. access to high-quality amenities, like upper-tier hospitals, in the elderly population
Guo et al. 2020 [27]	Framework for transportation equity assessment integrating accessibility, traffic emissions and safety outcomes.<br>The framework consists of three steps: Population measurement, Cost/benefit measurement and Inequality measurement. The framework is designed to assist researchers and transportation planners with equity analysis methods.	P	General population	Transportation	-	-	(D) = mention of the fairness of the distribution of impacts among populations and the distribution of outcomes among all individuals or among spatially distributed population groups (horizontal equity) vs. between population groups defined by demographic characteristics (vertical equity, e.g. race, ethnicity, income, education level)
Ingram et al. 2020 [28]	Framework for achieving transportation equity through the lens of critical race theory. Considering community engagement and centering the margins, social location and race consciousness.	P, I, (E)	Vulnerable and marginalized populations	Transportation	Tucson, USA, one of the poorest and most economically segregated cities in the country	-	(D) = mention of a political need of fairness in resource distribution across the city and in society<br>(P) = mention of existing practices to get public feedback often not reaching the most affected or vulnerable population groups – therefore a model of future collaborative community engagement

							was provided for the future to involve those that have not been as historically engaged or had the opportunity to be engaged<br>(R) = mention of the importance of prioritizing marginalized persons, highlighting that experiences of and responses to marginalization are not uniform – therefore efforts were made to include and privilege marginalized perspectives, e.g. through strategies like digital stories and workshops or through community-driven efforts
Lamorgese & Geneletti 2013 [29]	Framework for analysing the degree of consideration of sustainability principles in Strategic environmental assessment (SEA), consisting of the following 7 principles: (1) Precaution and adaptation, (2) Inter-generational equity, (3) Ecological integrity and biological diversity, (4) Equity and quality of life, (5) Efficiency, (6) Democracy and governance, (7) Immediate and long-term integration. Criteria and questions are given for each principle.	P, I	General population living in larger cities (50,000+ inhabitants) in Italy	(Strategic environmental assessment)	Environmental reports of 15 larger cities (50,000+ inhabitants) in Italy	-	(D) = mention of the fairness of distribution of environmental goods and bads and exposition to health risks – questions focussing on equity of exposure, equity of impact (in which groups should experience a proportionate share of costs and benefits of a change) and avoiding inequities in the impacts of environmental policies (differences in regulatory enforcements, e.g. sectors

							of communities who are imposed additional costs and the increased price of certain goods)<br>(P) = mention of active public participation for a transparent decision-making process – questions regarding avoiding inequities in people's ability to influence decisions affecting their environment and assuring that all members of the public have the opportunity to comment on proposals and have their views taken into account before decisions are being made
Meerow et al. 2019 [9]	Conceptualising social equity in the context of urban resilience planning: Distributive, Recognitional and Procedural Justice. Qualitative coding scheme for resilience strategy documents. The aim is to inform theoretical and empirical debates by examining how social equity is incorporated into resilience planning.	P	General population in cities in North America	(resilience strategies)	First ten North American city resilience plans created through the 100RC programme.	-	(D) = highlighted as a part of the conceptual model for social equity in the context of urban resilience planning - focussing on goods and infrastructure, environmental (dis)amenities, services and opportunities<br>(P) = highlighted as a part of the conceptual model for social equity in the context of urban resilience planning, focussing on participation

							in plan development, participation in governance and outreach to marginalized groups (R) = highlighted as a part of the conceptual model for social equity in the context of urban resilience planning, focussing on recognizing group history and needs and promoting respect
Okamoto & Doyon 2024 [30]	JustAdapt: Evaluation framework for urban coastal adaptation planning (UCAP); six-step implementation process; best utilized when applied within a larger UCAP process and after pre-work on equity, decolonization and equitable UCAP; helpful for practitioners to reflect upon and shift equity and justice within their own contexts; adaptable for different climate and environmental hazards	P, I, E	General population, with a special focus on equity-denied populations	(urban coastal adaptation planning)	-	-	Clear mention of and focus on justice aspects within the publication and the framework: (D), (P), (R) = Definition, considerations, opportunity and related questions given within the framework; additionally, intergenerational justice (planning guided by generational thinking) and epistemic justice (knowledge of indigenous and other equity-denied populations respected and valued)
Walton & Emmanuel (2010) [31]	The assessment framework: Assessing the dynamics of environmental inequity and distributive environmental equity. Seven framework steps divided in scoping level assessment (to establish the	(P)	General population in the UK	Five key environmental equity issues: noise impacts, air-quality impacts, visual impacts,	-	-	(D) = mention of the distribution of environmental impacts among disadvantaged groups and the possibility of a project's likely environmental benefits

	need for, and coverage of, any main assessment by determining which environmental equity issues are relevant to the proposed development) and main assessment (to determine the extent of the possible environmental equity (or inequity) associated with the proposed development). The framework is designed for decision makers to examine the environmental equity implications of proposed urban developments.			community severance impacts, property and community facility impacts			and burdens among those considered deprived and not deprived, with a mention of key environmental equity issues/impacts: noise, air-quality, visual, community severance, property and community facility;<br>(P) = mention of the definition of "Procedural equity" (i.e. equity in impacting upon a decision) highlighting the different opportunity, capacity and leverage different groups have to participate in decisions affecting their environment
World Health Organization (2010) [32]	Urban HEART: Urban Health Equity Assessment and Response Tool – a guide for policy- and decision-makers to identify and analyse inequities in health and facilitate decisions on viable and effective strategies, interventions and actions to reduce health inequities. The three core elements of URBAN HEART are: sound evidence, intersectoral action for health and community participation.	P, I, E	General population, but especially focused on disadvantaged population groups.	-	-	x	(D) = mention of systematic differences in health that are not distributed randomly, especially systematic differences in health between different socioeconomic groups<br>(P) = Community participation is mentioned as one of the core elements of Urban HEART; mention of systematic exclusion of some population groups from participation in decision making processes, e.g.

							gender and ethnicity-related barriers to participation in decision-making result in unequal access to resources, capabilities and rights
