# Supplementary material for: A new framework to consider equity in urban intervention planning, implementation and evaluation: development and application in a case study on an urban play spaces policy
Source: BMC Public Health. 2026 Feb 24;26:721. doi: 10.1186/s12889-026-26449-7 (PMC12930818; doi:10.1186/s12889-026-26449-7)
Supplement: Supplementary file 4 — Supplementary Material 4. Search terms. Search terms for literature on play spaces. [file 12889_2026_26449_MOESM4_ESM.pdf]

Additional File 4

Search terms – Play spaces

Block A

Search name	Search query	Type of search
A1	(TITLE-ABS-KEY(playground)) OR (TITLE-ABS-KEY("play* area*")) OR (TITLE-ABS-KEY("play* corner*")) OR (TITLE-ABS-KEY("play* field*")) OR (TITLE-ABS-KEY(playable)) OR (TITLE-ABS-KEY("play* space*")) OR (TITLE-ABS-KEY("play* opportunit*")) OR (TITLE-ABS-KEY("play* facilit*")) OR (TITLE-ABS-KEY("play* place*")) OR (TITLE-ABS-KEY("play* offer*")) OR (TITLE-ABS-KEY("recreational area*")) OR (TITLE-ABS-KEY("recreational facilit*")) OR (TITLE-ABS-KEY("recreational opportunit*")) OR (TITLE-ABS-KEY("recreational space*")) OR (TITLE-ABS-KEY(park)) OR (TITLE-ABS-KEY(parks)) OR (TITLE-ABS-KEY("leisure facilit*")) OR (TITLE-ABS-KEY("leisure opportunit*")) OR (TITLE-ABS-KEY("leisure space*")) OR (TITLE-ABS-KEY("leisure area*"))	Title/abstract keywords
A2	(TITLE-ABS-KEY(neighbourhood*)) OR (TITLE-ABS-KEY(neighborhood*))	

Block B

Search name	Search query	Type of search
B	(TITLE-ABS-KEY(equit*)) OR (TITLE-ABS-KEY(equality)) OR (TITLE-ABS-KEY("socioeconomic difference*")) OR (TITLE-ABS-KEY("socio-economic difference*")) OR (TITLE-ABS-KEY("sociodemographic difference*")) OR (TITLE-ABS-KEY("socio-demographic difference*")) OR (TITLE-ABS-KEY("socioenvironmental difference*")) OR (TITLE-ABS-KEY("socio-environmental difference*")) OR (TITLE-ABS-KEY("socioeconomic gradient")) OR (TITLE-ABS-KEY("socio-economic gradient")) OR (TITLE-ABS-KEY("social gradient")) OR (TITLE-ABS-KEY(inclusion)) OR (TITLE-ABS-KEY(inclusiv*)) OR (TITLE-ABS-KEY(opportunit*)) OR (TITLE-ABS-KEY(inequit*)) OR (TITLE-ABS-KEY(access*)) OR (TITLE-ABS-KEY(inequalit*))	Title/abstract keywords

Block C

Search name	Search query	Type of search
C	(TITLE-ABS-KEY(child*)) OR (TITLE-ABS-KEY(adolescen*)) OR (TITLE-ABS-KEY(youth)) OR (TITLE-ABS-KEY(teenage*))	Title/abstract keywords

Block D

Search name	Search query	Type of search
D	(TITLE-ABS-KEY(Netherlands)) OR (TITLE-ABS-KEY(Holland))	Title/abstract keywords

Search in Scopus
A1 AND B AND C AND D<br>with limitations „article“, „english“ and „2000-2022“
