# Supplementary material for: A new framework to consider equity in urban intervention planning, implementation and evaluation: development and application in a case study on an urban play spaces policy
Source: BMC Public Health. 2026 Feb 24;26:721. doi: 10.1186/s12889-026-26449-7 (PMC12930818; doi:10.1186/s12889-026-26449-7)
Supplement: Supplementary file 5 — Supplementary Material 5. Logic model of the intervention-specific framework for the Play Spaces case study, detailed for the phases planning, implementation and evaluation. Three figures showing details of the logic model for the Play Spaces case study, Fig. S1 planning phase, Fig. S2 implementation phase, Fig. S3 evaluation phase. [file 12889_2026_26449_MOESM5_ESM.pdf]

Additional File 5

Logic model of the intervention-specific framework for the Play Spaces case study, detailed for the phases planning, implementation and evaluation

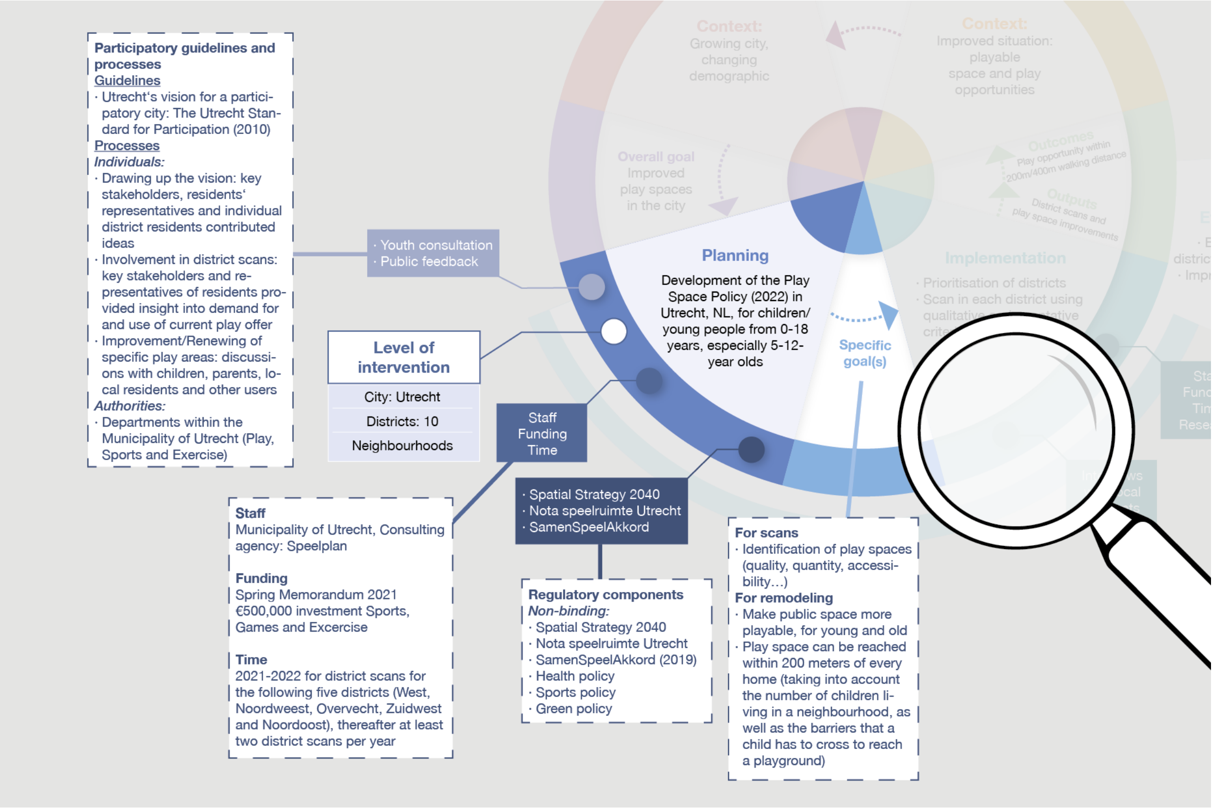

Fig. S1: Logic model of the intervention-specific framework - Play Spaces case study - Planning phase

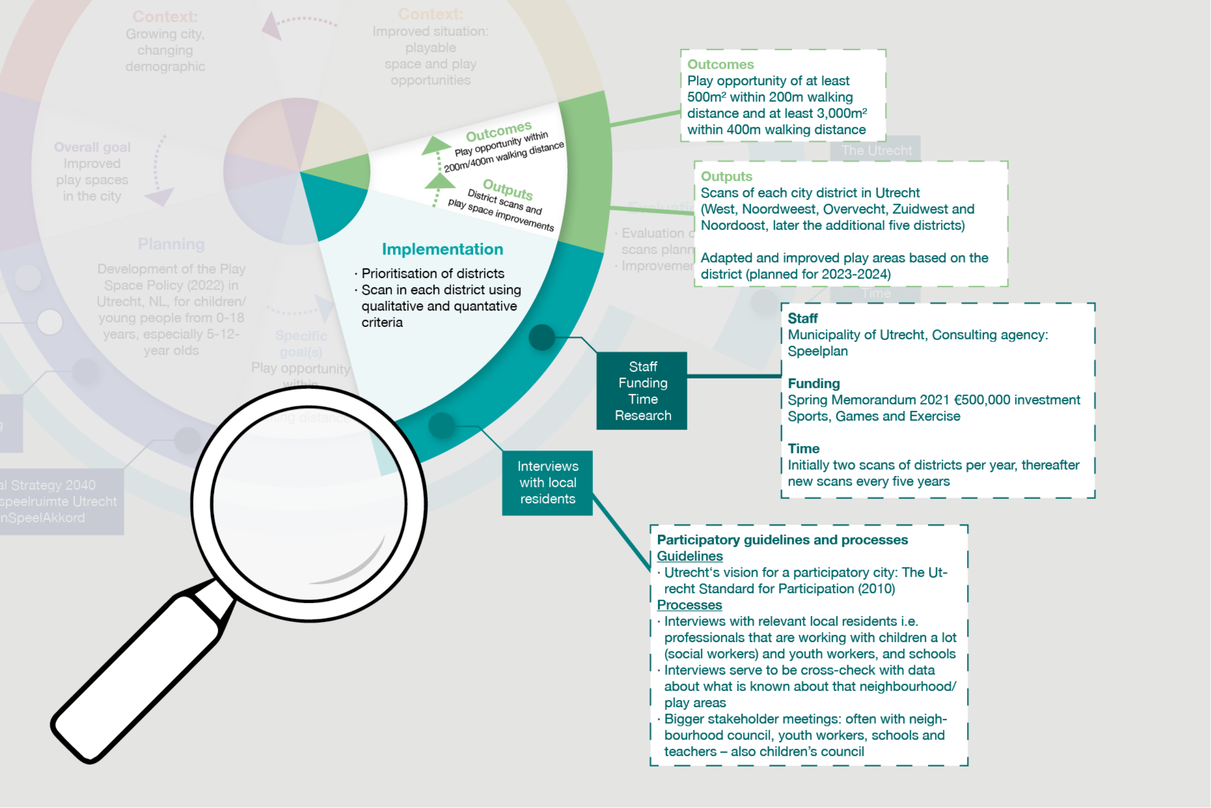

Fig. S2: Logic model of the intervention-specific framework - Play Spaces case study - Implementation phase

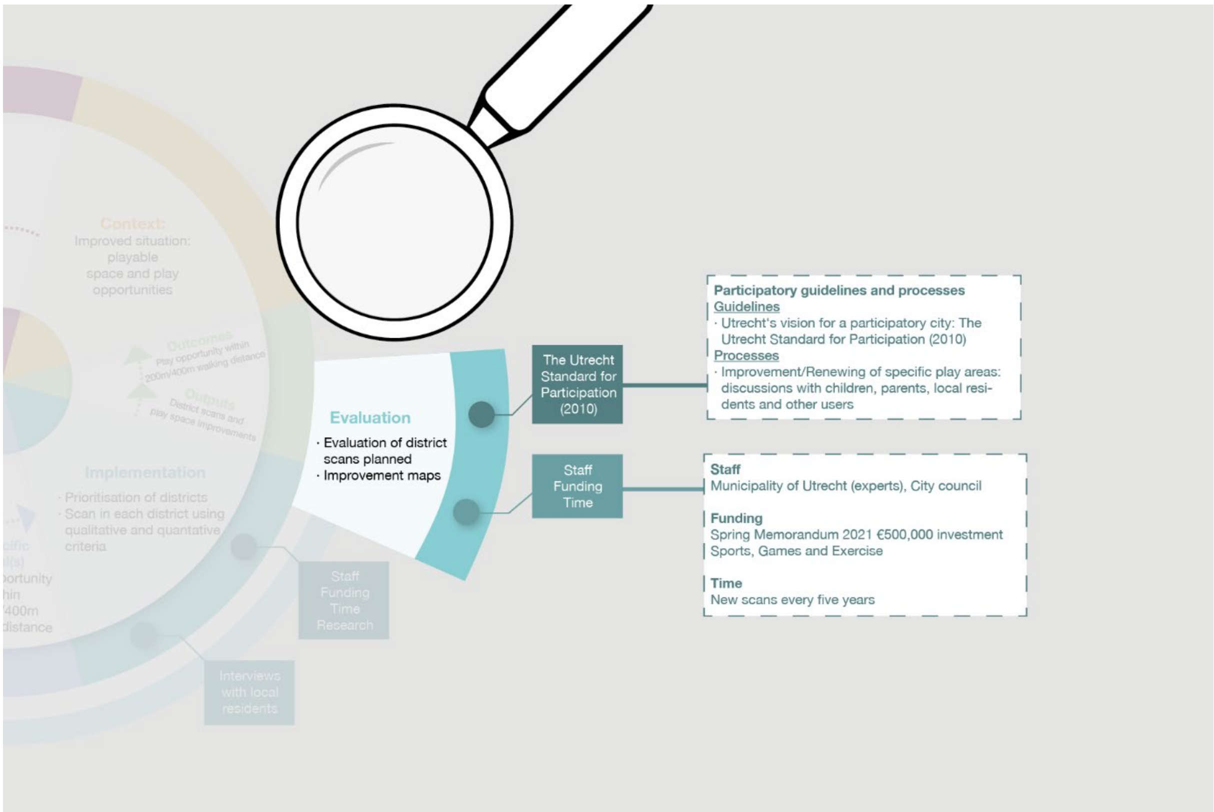

Fig. S3: Logic model of the intervention-specific framework - Play Spaces case study - Evaluation phase
