# Supplementary material for: A new framework to consider equity in urban intervention planning, implementation and evaluation: development and application in a case study on an urban play spaces policy
Source: BMC Public Health. 2026 Feb 24;26:721. doi: 10.1186/s12889-026-26449-7 (PMC12930818; doi:10.1186/s12889-026-26449-7)
Supplement: Supplementary file 6 — Supplementary Material 6. The Early environmental quality and life-course mental health effects (Equal-Life) project team. [file 12889_2026_26449_MOESM6_ESM.pdf]

Additional File 6

The Early environmental quality and life-course mental health effects (Equal-Life) project team

Kathryn Adams	Centre for Environmental Health and Sustainability & School of Geography, Geology and the Environment, University of Leicester, Leicester, United Kingdom
Aleksei Afonin	A.I.Virtanen Institute for Molecular Sciences, University of Eastern Finland, Kuopio, Finland
Jenny Ahrens	Institute of Public Health and Nursing Research, University of Bremen, Bremen, Germany
Marco Balduini	Quantia Consulting srl, Milan, Italy
Christin Belke	Centre for Applied Psychology, Environmental and Social Research (Zeus GmbH), Hagen, Germany
Sarah Leona Benz	Centre for Applied Psychology, Environmental and Social Research (Zeus GmbH), Hagen, Germany
Anneke Blokstra	Centre for Nutrition, Prevention and Health Services, National Institute for Public Health and the Environment, Bilthoven, The Netherlands
Sandra Boekhold	Centre for Sustainability, Environment and Health, National Institute for Public Health and the Environment, Bilthoven, The Netherlands
Jolanda Boer	Centre for Nutrition, Prevention and Health Services, National Institute for Public Health and the Environment, Bilthoven, The Netherlands
Rik Bogers	Centre for Sustainability, Environment and Health, National Institute for Public Health and the Environment, Bilthoven, The Netherlands
Sven Bölte	Unit of Occupational Medicine at the Institute of Environmental Medicine, Karolinska Institutet, Stockholm, Sweden
Gabriele Bolte	Institute of Public Health and Nursing Research, University of Bremen, Bremen, Germany
Hendriek Boshuizen	Centre for Nutrition, Prevention and Health Services, National Institute for Public Health and the Environment, Bilthoven, The Netherlands
Núria Botella	Barcelona Institute for Global Health, ISGlobal, Barcelona, Spain
Dick Botteldooren	Department of Information Technology, Faculty of Engineering and Architecture, Ghent University, Ghent, Belgium
Alessandro Bozzon	Department of Sustainable Design Engineering, Faculty of Industial Design Engineering, Delft University of Technology, Delft, The Netherlands
Ella Braat-Eggen	University of Applied Science, Eindhoven, Netherlands
Marco Brambilla	Quantia Consulting srl, Milan, Italy
YingXin Chen	Centre for Environmental Health and Sustainability & School of Geography, Geology and the Environment, University of Leicester, Leicester, United Kingdom
Yun Chen	School of Public Health and Community Medicine, Gothenburg University, Sweden
Boris Cheval	Barcelona Institute for Global Health, ISGlobal, Barcelona, Spain

Charlotte Clark	St George's, University of London, United Kingdom
Payam Dadvand	Barcelona Institute for Global Health, ISGlobal, Barcelona, Spain
Nele De Poortere	Department of Information Technology, Faculty of Engineering and Architecture, Ghent University, Ghent, Belgium
Luc Dekoninck	Department of Information Technology, Faculty of Engineering and Architecture, Ghent University, Ghent, Belgium
Maud Dohmen	Built Environment, Technical University Eindhoven, Eindhoven, The Netherlands
Gerda Doornbos	Centre for Nutrition, Prevention and Health Services, National Institute for Public Health and the Environment, Bilthoven, The Netherlands
Gabin Drouard	Institute for Molecular Medicine Finland, University of Helsinki, Helsinki, Finland
Angel Dzhambov	Institute for Highway Engineering and Transport Planning, Graz University of Technology, Graz, Austria
Michail Evangelos Terzakis	Built Environment, Technical University Eindhoven, Eindhoven, The Netherlands
Janina Fels	Institute for Hearing Technology and Acoustics (IHTA), RWTH Aachen University, Aachen, Germany
Christoph Giehl	RPTU, Rheinland-Pfälzische Technische Universität, Kaiserslautern, Germany
Helene Gudi-Mindermann	Institute of Public Health and Nursing Research, University of Bremen, Bremen, Germany
John Gulliver	St George's, University of London, United Kingdom
Anna Hansell	Centre for Environmental Health and Sustainability & School of Geography, Geology and the Environment, University of Leicester, Leicester, United Kingdom
Marja Heinonen-Guzejev	Institute for Molecular Medicine Finland,University of Helsinki, Helsinki & Department of Public Health University of Helsinki, Helsinki, Finland
Jurriaan Hoekstra	Center for Sustainability, Environment and Health, National Institute for Public Health and the Environment, Bilthoven, The Netherlands
Maarten Hornikx	Built Environment, Technical University Eindhoven, Eindhoven, The Netherlands
Sammie Jansen	Centre for Sustainability, Environment and Health, National Institute for Public Health and the Environment, Bilthoven, The Netherlands
Calvin Jephcote	Centre for Environmental Health and Sustainability & School of Geography, Geology and the Environment, University of Leicester, Leicester, United Kingdom
Sonja Jeram	National Institute of Public Health, Ljubljana, Slovenia
Jordi Julvez	Pere Virgili Institute for Health Research, Barcelona, Spain
Katja Kanninen	A.I.Virtanen Institute for Molecular Sciences, University of Eastern Finland, Kuopio, Finland
Jaakko Kaprio	Institute for Molecular Medicine Finland, University of Helsinki, Helsinki & Department of Public Health University of Helsinki, Helsinki, Finland
Maria Klatte	RPTU, Rheinland-Pfälzische Technische Universität, Kaiserslautern, Germany

Mihail Kochubovski	Institute of Public Health of the Republic of North Macedonia, Skopje, North Macedonia
Gerd Kortuem	Department of Sustainable Design Engineering, Faculty of Industial Design Engineering, Delft University of Technology, Delft, The Netherlands
Julia Kuhlmann	Centre for Applied Psychology, Environmental and Social Research (Zeus GmbH), Hagen, Germany
Thomas Lachmann	RPTU, Rheinland-Pfälzische Technische Universität, Kaiserslautern, Germany
Larissa Leist	RPTU, Rheinland-Pfälzische Technische Universität, Kaiserslautern, Germany
Peter Lercher	Institute for Highway Engineering and Transport Planning, Graz University of Technology, Graz, Austria
Karin Loh	Hearing Technology and Acoustics, RWTH Aachen University, Aachen, Germany
Jesper Löve	School of Public Health and Community Medicine, Gothenburg University, Gothenburg, Sweden
Sanne Meijering	Centre for Sustainability, Environment and Health, National Institute for Public Health and the Environment, Bilthoven, The Netherlands
Vasileios Milias	Department of Sustainable Design Engineering, Faculty of Industial Design Engineering, Delft University of Technology, Delft, The Netherlands
Kerstin Persson Waye	School of Public Health and Community Medicine, Gothenburg University, Gothenburg, Sweden
Carmen Peuters	Barcelona Institute for Global Health, ISGlobal, Barcelona, Spain
Aino-Kaisa Piironen	A.I.Virtanen Institute for Molecular Sciences, University of Eastern Finland, Kuopio, Finland
Achilleas Psyllidis	Department of Sustainable Design Engineering, Faculty of Industial Design Engineering, Delft University of Technology, Delft, The Netherlands
Andrei Pyko	Unit of Occupational Medicine at the Institute of Environmental Medicine, Karolinska Institutet, Stockholm, Sweden
Bruno Raimbault	Barcelona Institute for Global Health, ISGlobal, Barcelona, Spain
Gordana Ristovska	Institute of Public Health of the Republic of North Macedonia, Skopje, North Macedonia
Ioar Rivas	Barcelona Institute for Global Health, ISGlobal, Barcelona, Spain
Jana Roczen	Institute of Public Health and Nursing Research, University of Bremen, Bremen, Germany
Nestor Sanchez Martinez	Unit of Occupational Medicine at the Institute of Environmental Medicine, Karolinska Institutet, Stockholm, Sweden
Anna Sandionigi	Quantia Consulting srl, Milan, Italy
Dirk Schreckenberg	Centre for Applied Psychology, Environmental and Social Research (Zeus GmbH), Hagen, Germany
Julia Seitz	Institute for Hearing Technology and Acoustics (IHTA), RWTH Aachen University, Aachen, Germany
Jenny Selander	Unit of Occupational Medicine at the Institute of Environmental Medicine, Karolinska Institutet, Stockholm, Sweden
Michael Smith	School of Public Health and Community Medicine, Gothenburg University, Gothenburg, Sweden

Jan Spilski	RPTU, Rheinland-Pfälzische Technische Universität, Kaiserslautern, Germany
Igor Spiroski	Institute of Public Health of the Republic of North Macedonia, Skopje, North Macedonia
Libor Sulc	Unit of Occupational Medicine at the Institute of Environmental Medicine, Karolinska Institutet, Stockholm, Sweden
Wim Swart	Centre for Sustainability, Environment and Health, National Institute for Public Health and the Environment, Bilthoven, The Netherlands
Roos Teeuwen	Department of Sustainable Design Engineering, Faculty of Industial Design Engineering, Delft University of Technology, Delft, The Netherlands
Justus Tönnies	Institute of Public Health and Nursing Research, University of Bremen, Bremen, Germany
Peter van den Hazel	International Network for Children's Health, Environment and Safety (INCHES)
Irene van Kamp	Centre for Sustainability, Environment and Health, National Institute for Public Health and the Environment, Bilthoven, The Netherlands
Elise van Kempen	Centre for Sustainability, Environment and Health, National Institute for Public Health and the Environment, Bilthoven, The Netherlands
Timothy van Renterghem	Department of Information Technology, Faculty of Engineering and Architecture, Ghent University, Ghent, Belgium
Harm van Wijnen	Centre for Sustainability, Environment and Health, National Institute for Public Health and the Environment, Bilthoven, The Netherlands
Sarah Verhulst	Department of Information Technology, Faculty of Engineering and Architecture, Ghent University, Ghent, Belgium
Markus Viljanen	Centre for Nutrition, Prevention and Health Services, National Institute for Public Health and the Environment, Bilthoven, The Netherlands
Natalia Vincens	School of Public Health and Community Medicine, Gothenburg University, Gothenburg, Sweden
Tanja Vrijkotte	Department of Public and Occupational Health, Amsterdam Public Health Research Institute, The Netherlands
Zhiyang Wang	Institute for Molecular Medicine Finland, University of Helsinki, Helsinki, Finland
Miriam Weber	Municipality of Utrecht, The Netherlands
Alyce Whipp	Institute for Molecular Medicine Finland, University of Helsinki, Helsinki, Finland
Kim White	Centre for Sustainability, Environment and Health, National Institute for Public Health and the Environment, Bilthoven, The Netherlands
Maddie White	Institute of Public Health and Nursing Research, University of Bremen, Bremen, Germany
Albert Wong	Centre for Nutrition, Prevention and Health Services, National Institute for Public Health and the Environment, Bilthoven, The Netherlands
